# Exchanging and using research evidence in health policy networks: a statistical network analysis

**DOI:** 10.1186/s13012-014-0126-8

**Published:** 2014-10-30

**Authors:** Jessica C Shearer, Michelle Dion, John N Lavis

**Affiliations:** Department of Clinical Epidemiology and Biostatistics, Centre for Health Economics and Policy Analysis, McMaster University, Hamilton, ON Canada; Department of Political Science, McMaster University, Hamilton, ON Canada

**Keywords:** Evidence-informed health policy, Knowledge translation, Social network analysis, Exponential random graph model, Policy network

## Abstract

**Background:**

Evidence-informed health policymaking is a goal of equitable and effective health systems but occurs infrequently in reality. Past research points to the facilitating role of interpersonal relationships between policy-makers and researchers, imploring the adoption of a social network lens. This study aims to identify network-level factors associated with the exchange and use of research evidence in policymaking.

**Methods:**

Data on social networks and research use were collected from seventy policy actors across three health policy cases in Burkina Faso (child health, malaria, and HIV). Networks were graphed for actors’ interactions, their provision of, and request for research evidence. Exponential random graph models estimated the probability of evidence provision and request between actors, controlling for network- and individual-level covariates. Logistic regression models estimated actors’ use of research evidence to inform policy.

**Results:**

Network structure explained more than half of the evidence exchanges (ties) observed in these networks. Across all cases, a pair of actors was more likely to form a provision tie if they already had a request tie between them and visa versa (*θ* = 6.16, *p* < 0.05; *θ* = 2.87, *p* < 0.05; *θ* = 2.31, *p* < 0.05). The child health network displayed clustering tendencies, meaning that actors were more likely to form ties if they shared an acquaintance (*θ* = 2.36, *p* < 0.05). Actors’ use of research evidence was positively associated with their centrality (*i.e.*, connectedness).

**Conclusions:**

The exchange and use of research evidence in policymaking can be partly explained by the structure of actors’ networks of relationships. Efforts to support knowledge translation and evidence-informed policymaking should consider network factors.

**Electronic supplementary material:**

The online version of this article (doi:10.1186/s13012-014-0126-8) contains supplementary material, which is available to authorized users.

## Background

The use of research evidence to inform health policy decision-making has been identified as a means to improve the effectiveness of health policy decisions [1-3], to strengthen health systems [4], and to achieve universal health coverage [5]. Yet, evidence-informed policymaking occurs relatively infrequently for reasons related to the evidence itself [6], the policy issue being discussed [7], and because evidence ‘competes’ with many other inputs in a complex policy-making environment, including institutions, interests, ideas and external events [1,3]. There is a growing understanding among stakeholders of the complexity of evidence-informed policymaking and thus the need for approaches that understand and address such complexity [8].

The literature on evidence exchange and use in health policymaking [3,8-11] consistently identifies interpersonal relationships between researchers and policy-makers as having a positive effect [8,12-16]. Developing relationships between producers and users has become a core focus of knowledge translation interventions [16,17], operationalized into approaches such as ‘knowledge brokers’ and ‘deliberative dialogues’ [18]. Justifications for such strategies include arguments related to shifting the incentives for research producers [17], reducing the costs to research users [8], reducing conflict around research evidence [17], and facilitating trust [8,17]—all of which are thought to increase the likelihood of exchange and use. Yet, few of these approaches explain why some relationships form and others do not, beyond the simplistic ‘research producer’ versus ‘research user’ dichotomy. Even deliberative dialogues, which succeed in bringing together a range of actors, and thus affect network structure, operate with limited understanding of the social structure they are intervening on, or the causal mechanisms underlying their intervention. Thus, despite calls for more attention to social structure and social networks [8,19], little has been done to elucidate the causal mechanisms underlying the current field of socially-targeted interventions for evidence-informed health policy.

This study applies an explicit social network approach in order to answer two research questions related to evidence exchange and use in health policymaking: first, what factors are associated with the formation of research exchange relationships between policy actors; and second, to what extent are these exchange relationships associated with the use of research evidence by policy actors in the policymaking process? These results will have important implications for the design of interventions for knowledge translation and evidence-informed health policymaking.

### Social network analysis as a framework to understand evidence-informed health policy

Social network analysis (SNA) is a theory as well as a set of tools for exploring socially-influenced behaviours. Social network theory offers a persuasive explanation of why evidence exchange and use should be understood from an interpersonal, as well as structural, perspective. As seen through a social network lens, behaviours—in this case, evidence exchange and use—are predicted not only by an individual’s attributes, but by his or her position in her social environment, and the larger structure of that environment. Social network analysis has recently been applied to evidence exchange in a municipal public health department [20] and to information, broadly speaking, within organizations [21-25] and political networks, [13] but never to evidence exchange and use in a national policy setting. We thus extend the existing research agenda to answer questions about the exchange of research evidence (the social process) within policy networks (the social setting). Policy networks are simply social networks specific to policy actors—defined here as sets of individuals who interact on a given policy issue, and may include a range of actors from various sectors and levels of governance. In contrast to formal organograms or stakeholder maps that document who are expected to (or should) participate according to organizational boundaries [26], policy networks are empirically-driven measurements of who actually participates, thus embracing a policy arena’s fluidity and diversity. In many settings, including low- and middle-income countries, policy decisions are made by a range of diverse actors with varying levels of formal and informal power [27-29].

While social network analysis can be applied to research questions at actor, tie, or network levels, this study specifically exploits recent methodological innovations in statistical network analysis enabling the prediction of ties between pairs of actors as some function of their individual attributes and network structure. These models are referred to as the exponential random graph class of models (ERGM) [30,31]. The present paper uses ERGMs to model the existence of research evidence exchange ties, specifically evidence provision and request, in a policy network.

**Figure 1:**
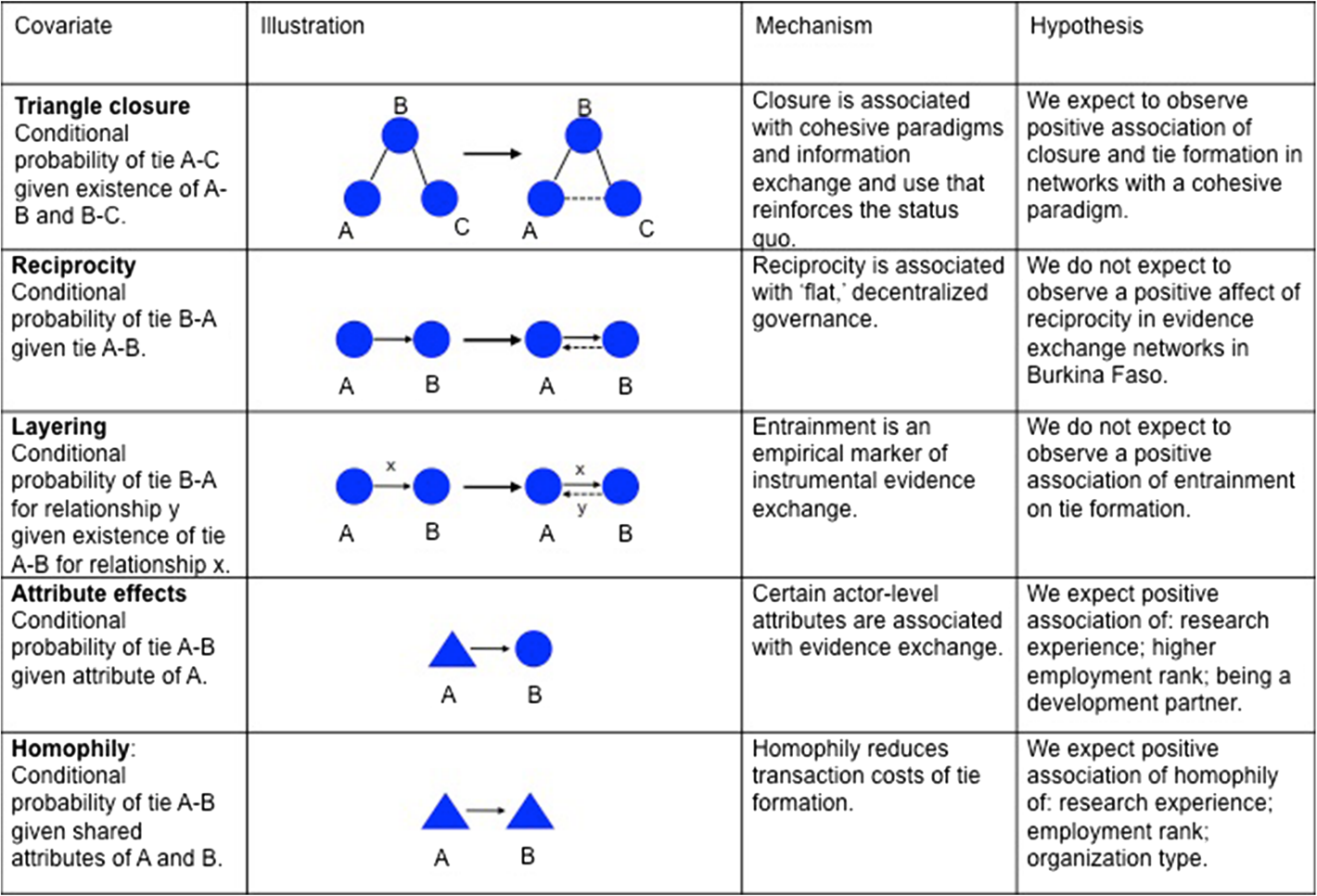
**Network covariates and their hypotheses.**

Ties can form, in theory, between any two individuals. But in practice, network scientists observe more frequent tie formation in the presence of certain network structures. The next section presents common network hypotheses adjusted to the context of Burkina Faso (see Figure 1 for a synthesis), where, as in many low-income countries, the formal culture of research production, access and use is relatively weak due to language barriers, poor Internet access, and general resource limitations [32,33]. A better understanding of interpersonal modes of exchange will be highly relevant for Burkina Faso and other low-income countries.

First, individuals are more likely than chance alone to form ties with other individuals if they have a friend in common [34]. This phenomenon is referred to as triangle closure and looks like a triangle between three actors on a network graph (see Figure 1). In the policy science and governance literature, triangle closure is associated with stronger group cohesion, cooperation, and shared norms [35-37], and many redundant ties is associated with the fast diffusion of ideas amongst dyads and sub-groups. At the same time, triangle closure limits ‘structural holes’ in a network; that is, the areas of a network where actors are relatively unconnected [34]. It is the bridging of these structural holes that is associated with the efficient exposure to and diffusion of innovations and new ideas at the macro-level [35]. We expect to observe a positive association between triangle closure and tie formation in evidence exchange networks with cohesive cognitive paradigms. A network with a tendency for closure will likely exchange ideas that reinforce the status quo at the dyadic and sub-group level, but will limit the introduction and exchange of new ideas at the macro level.

‘Reciprocity’ is another social process that is frequently observed more often than chance alone [36,38]. For example, a request for evidence from Person A to Person B is reciprocated when Person B requests evidence from Person A (see Figure 1). Networks with high reciprocity are associated with ‘flat,’ or non-hierarchical, governance [39]. An evidence exchange network with high reciprocity would indicate that its actors have relatively similar levels of expertise, access to and demand for evidence, and the political capital to exchange it with each other, as was recently observed in a Canadian municipal public health department [20]. In contrast, policy actors in Burkina Faso have varying levels of capacity for finding and using research evidence and the policymaking culture is hierarchical, with power and expertise centralized among certain few actors. For these reasons, we do not expect to observe a positive association between reciprocity and tie formation in these networks.

‘Layering’ is the other side of the coin (also known as multiplexity or entrainment). Instead of measuring the existence of two ties for the same relation between a pair of actors, layering measures the co-existence of two different relations between the same pair of actors [36]. For example, imagine that Person A requests evidence from Person B. Layering occurs when Person B *provides* evidence to Person A (see Figure 1). Research exchange networks with high layering are achieving their instrumental, or practical, purpose—research is provided when requested—and we suggest that layering is an empirical signature of true ‘exchange’ models of knowledge translation [17]. A lack of layering could indicate many things. A tendency for provision ties to exist without request ties might indicate that evidence is being disseminated for advocacy purposes, and at the most extreme might signal the symbolic or political use of evidence [40]. On the other hand, a tendency for request ties to exist without provision ties might suggest that no evidence is available to fulfill the request, or that actors are refusing or ignoring requests. Both outcomes suggest a poor climate for research evidence. We anticipate that research is provided by a small group of development partners and researchers in this context, often in the absence of requests, and thus we do not expect to observe significant layering.

In addition to these structural effects, we assume that the individual attributes of actors will influence their propensity to provide or request research evidence. There have been few studies exploring which individual characteristics influence evidence exchange, other than to say that evidence is more likely to be exchanged by a researcher or someone with research experience [12]. We hypothesize that in this context, where the culture of research and evidence use is nascent, the provision and request of research evidence will occur more often by actors with research experience, actors with higher employment rank, and development partners. Each of those actor types will have greater resources and technical skills related to finding, exchanging and using research evidence. Higher employment rank and development partners may also signal exposure to larger external networks.

Finally, the phenomenon of *homophily* specifies that actors are more likely to form ties with ‘like’ individuals [41]. Homophily reduces transaction costs associated with exchanging evidence but limits the wide and efficient dissemination of evidence that would occur if ties connected researchers to non-researchers, crossed organizational boundaries, and traversed job levels. We expect to observe homophily in these networks.

## Methods

### Data collection

Social network and demographic data were collected between October 2011 and March 2012 from policy actors active in one or more of three health policy issues in Burkina Faso: community integrated management of childhood illness; home management of malaria; and removal of user fees for antiretroviral treatment for human immunodeficiency virus (HIV); henceforth referred to by their substantive categories: child health; malaria; and HIV. Policy cases were sampled according to pragmatic reasons, including large enough networks to enable statistical analysis, as well as for their diversity on network structural variables of interest. High quality and locally relevant research evidence was available to inform each of these policy cases and each decision involved some amount of uncertainty that could have been addressed using research evidence, typical of many health policy decisions made by ministries of health in Sub-Saharan Africa. That said, Burkina Faso faces many of the same challenges in using evidence to inform policy as other countries and some challenges that are context-specific; namely, slow internet connection limiting searches for evidence, and a working language (French) that offers fewer research publications. Commonalities include the fact that evidence must compete with many other inputs in the policymaking process—including institution-, interest-, and idea-related factors, and that the overall number of researchers, and thus research capacity, tends to be fewer in low-income countries. A recent rise in donor applications requiring evidence of the problem and options has perhaps introduced incentives to exchange and use research evidence.

Burkina Faso is like other Sub-Saharan African countries in terms of level of development, political regime type, and dependence on foreign aid, as well as a health policy sector characterized by participation of diverse actors and frequent changes in institutions. These characteristics will improve the generalizability of results, contributing to knowledge translation efforts, and evidence-informed policymaking, in other Sub-Saharan African countries.

We defined policy actors as any individual who participated in policy formulation for each policy issue. Actors were identified according to established procedures for respondent-driven censuses of social networks [42]. Beginning with a review of policy documents and informational interviews with key informants, the lead researcher (JS) approached two actors from each policy issue considered to be central in their issue networks for an interview. The researcher asked: ‘With whom did you interact during policy formulation?’ generating ‘interaction’ ties. Respondents were encouraged to provide as many names as possible; respondents provided an average of five names. The researcher approached each actor named and the same process was carried out (see Additional file 1 for a consort diagram detailing this process). Following other studies of policy networks [43], we chose to cease sampling when a round elicited fewer new names compared to the previous round. This resulted in four rounds of nominations: most new nominations came during the second and third rounds; the fourth round consisted of 23 of a total of 116 nominations (19.8%) and actors interviewed during the fourth round nominated only six new actors. Of 101 unique actors identified, 69 were reached for an interview. Actors who participated in more than one issue were interviewed successively for each issue. Missing ties were dropped in the analysis.

Upon listing their interaction relationships, respondents were then asked to specify whether they had provided research evidence to any of the names they listed (provision ties), or requested research evidence from any of the names they listed (request ties). Ties were coded as 1 if at least one of the actor-pair reported that a tie existed. Provision and request ties were coded as directed from one actor to another based on each respondents’ reports of their exchanges and could occur in both directions. Provision and request ties could occur simultaneously (*i.e.*, layering), but were considered separate constructs, and as such could also exist in the absence of the other.

Data were collected on actors’ relevant individual attributes (see Table 1), including: ‘research experience,’ defined as any formal experience participating in or leading a research project; ‘organization type,’ differentiating between actors working for government, development partner organizations, civil society organizations (CSO) and/or non-government organizations (NGO), and others (research organizations or independent consultants); and employment rank, dichotomized into manager/director-level and higher, or not. In-depth interviews elicited respondents’ perceptions and understandings of the policy issues as well as their awareness and use of research evidence during the policy-making process. Social desirability bias was avoided by indirect lines of questioning that did not suggest that respondents’ use of research evidence was being measured and response validity was achieved through probing strategies and in asking for specific examples or citations if a respondent claimed to have been aware of research on the topic, or to have included evidence in their reports. We hypothesize that an actor’s score on the use scale will be positively associated with their connectedness in the network, or ‘degree,’ where out-degree counts the number of ties an actor sends and in-degree counts ties received. We expect to see the highest rates of use amongst actors who provide and request evidence more frequently (*i.e.*, high out-degree in either network) as the active sending of ties suggests positive attitudes related to evidence.

**Table 1:** **Descriptive statistics**

**Variables**	**Child health**	**Malaria**	**HIV**
*Actor variables*	Mean (sd) or n (%)	Mean (sd) or n (%)	Mean (sd) or n (%)
Total actors nominated in 4 rounds	39	49	40
Actors surveyed	21	30	19
Male	13 (68.4)	24 (80.0)	16 (76.2)
Graduate-level degree	17 (89.5)	27 (90.0)	18 (90.0)
Years in position	3.11 (2.85)	4.48 (4.06)	7 (4.87)
Experience as researcher	8 (44.4)	14 (46.7)	12 (57.1)
Government org	14 (73.7)	17 (56.7)	8 (38.1)
Civil society organization	0 (0)	9 (30.0)	9 (42.9)
Development partner organization	4 (21.1)	2 (6.67)	2 (9.52)
Other organization	1 (5.26)	2 (6.67)	2 (9.52)
Manager or higher employment level	9 (47.4)	8 (26.7)	13 (61.9)
*Research use outcomes*			
Research use (continuous)	3.50 (1.03)	2.69 (1.44)	2.89 (1.52)
Any research use (binary)	7 (43.8)	10 (34.6)	6 (31.6)
*Provision network*			
Edges	37	36	28
Mean degree	1.95	1.20	1.33
Density	0.11	0.04	0.07
Triangles	80	10	6
Reciprocated edges	6	1	2
Layered edges	16	20	8
*Request network*			
Edges	17	25	10
Mean degree	0.895	0.833	0.476
Density	0.05	0.03	0.02
Triangles	10	8	0
Reciprocated edges	1	1	1
Layered edges	16	20	8

Qualitative interviews were tape-recorded and transcribed and data were managed and coded using NVivo software. Ethical approval was received from McMaster University’s Faculty of Health Sciences Ethical Review Board and the Burkina Faso Ministry of Health Ethics Committee in Health Research. Signed consent was received from all study participants.

### Analysis

The probability of a tie existing between any two given actors is modeled using ERGMs, which can be expressed as a conditional log-odds of individual ties in a network:

1 $$ \mathbf{logit}\left(\mathbf{P}\left({Y}_{ij}=\mathbf{1}\Big|\mathbf{rest}\ \mathbf{of}\ \mathbf{network}\right)\right) = \boldsymbol{\uptheta}^{\prime}\boldsymbol{\Delta} {\left(g\left(\mathbf{y}\right)\right)}_{\mathbf{ij}} $$

where Yij is an actor pair in network Y, θ is the vector of coefficients, Δ(*g*(y))ij is the change in the vector of network statistics g(y) when the value of Yij changes from 0 to 1. As in other regression models, we can include covariates to control for other processes that might affect the existence of a tie. ERGMs are different from normal logistic models in that they can control for network structure, or structural effects (SE), as well as actor characteristics, or attribute effects (AE). Structural effects models (SE models) included parameters for triangle closure, reciprocity and layering. Triangle closure was modeled using the geometrically weighted edgewise shared partner distribution (GWESP) statistic, which has been shown to overcome model degeneracy by specifying decreasing marginal impact of the formation of triangles on tie formation. [26,35] AE include researcher experience, organization type, employment rank and homophily. SE and AE models were run separately and then aggregated into full models.

Provision and request ties, the dependent variables, were modeled separately for each of the three policy networks, conditional on the existence of an interaction tie. Covariates were each modeled separately to determine which were most significant according to p-values and significant covariates were then entered into the full model step-wise based on their *p-value* in the null model (most to least significant). Covariates were retained in the final model if they improved model fit as tested by Akaike Information Criterion (AIC) and likelihood ratio tests. ERGM coefficients represent the conditional log-odds of a tie. Data were managed using Microsoft Excel and analyzed in R using the Statnet suite of packages [44], including ‘ergm’ [45]. The ERGM model likelihood function was approximated using Markov chain Monte Carlo simulation methods [44].

Goodness of fit was tested by comparing simulated networks to the observed networks to determine how well the model could reproduce global network properties (see Additional file 2 for goodness-of-fit test results).

The determinants of an actor’s use of evidence were explored in logistic regression models where the dependent variable was evidence use and was derived from in-depth interviews. A validated scale of evidence use by policy-makers [46,47] was applied to interview data, assigning each actor a value from 0 (no evidence use) to 5 (‘I made efforts to use this research evidence in decisions related to this policy issue’) based on the qualitative analysis of respondents’ discussion of how they used evidence during the policy process. While we had hoped to explain the ordinal outcome of ‘evidence use’ in regression models, those models did not converge and each actor’s score was collapsed into a binary dependent variable where the fourth category (‘I cited the research evidence in my own professional reports, documents or conversations’) and fifth (‘I made efforts to use the research evidence in decisions related to this policy issue’) were coded as ‘use’ and the third category (‘I participated in meetings for discussion and dissemination of the research evidence’) and those below it were coded as ‘non-use.’ Univariate logistic regression models tested whether use was associated with in-degree and out-degree in the provision and request networks, and with actor attributes.

## Results

**Figure 2:**
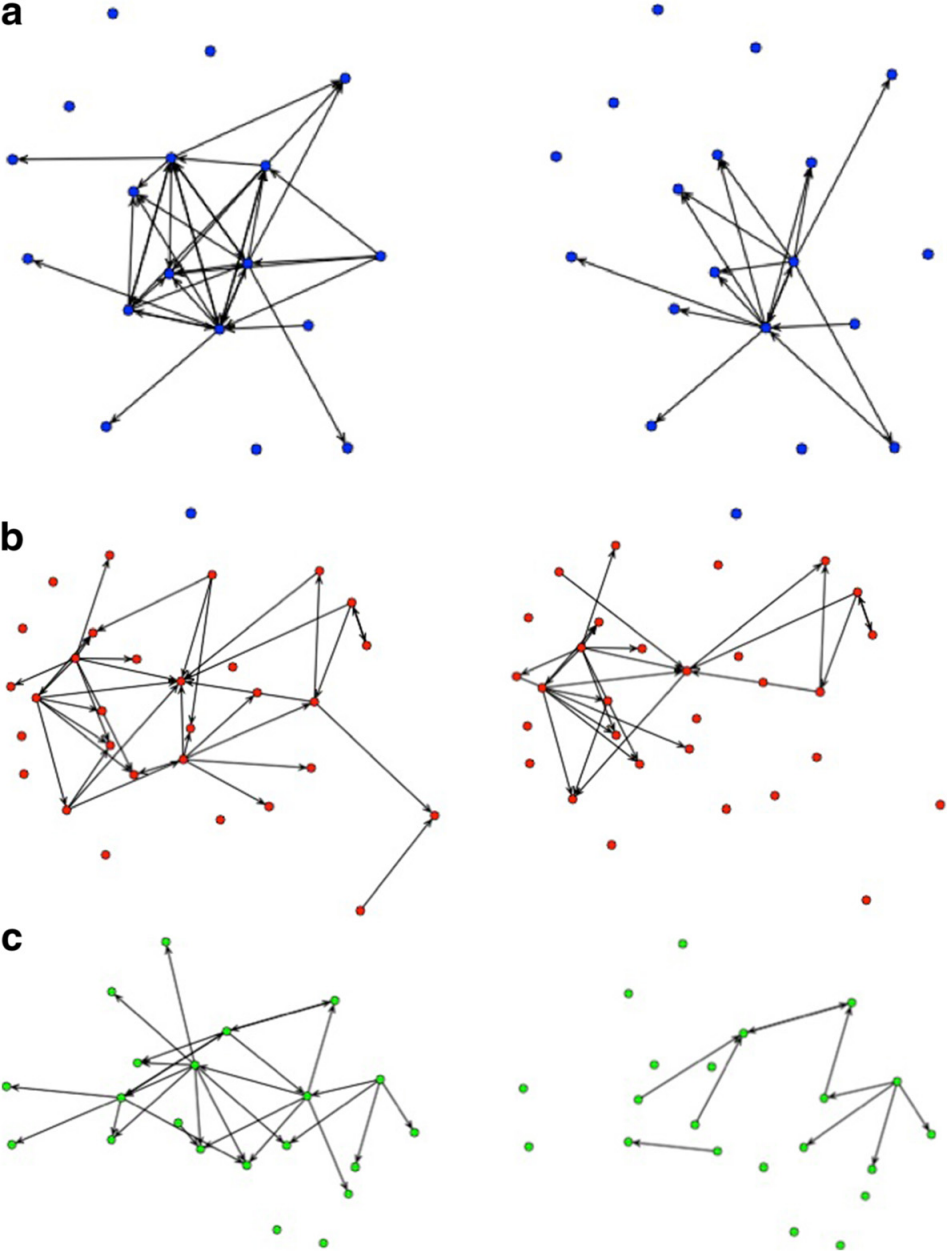
**Graphs of policy networks. (a)** Child health evidence provision (left) and evidence request (right) **(b)** Malaria evidence provision (left) and evidence request (right) **(c)** HIV evidence provision (left) and evidence request (right). Arrows indicate direction of relationship.

Table 1 describes the networks and their actors. Network composition varied slightly across issues, particularly in terms of organizational affiliation and employment level. Across all issues, provision networks were denser than request networks, meaning that evidence was provided more than requested. The child health networks were the densest and had the highest average degree, indicating that evidence exchange occurred more often for this issue than for the others. The child health networks had more triangles than other networks (80 triangles in the provision network). Approximately 13% of actors participated in more than one policy network. Figure 2 illustrates provision and request networks for each case.

### Why do policy actors exchange research evidence?

The provision and request of research evidence were associated with factors related to both structural and attribute effects. All model converged [44], although some covariates did not improve model fit as judged by AIC criteria and were thus excluded.

#### Provision models

Models for child health and malaria networks fit best when they combined structural and attribute effects (see Table 2 for full results). HIV models were slightly better fit when only modeling actor attributes. Actors were more likely to form provision ties if they also had a request tie (*i.e.*, layering), an effect that was statistically significant across issues. Only the child health network demonstrated a tendency for triangle closure (*θ* = 2.36; OR = 10.6, *p* < 0.05) where the odds of a provision tie were 10 times greater if that tie closed a triangle between three actors, conditional on the rest of the model. This suggests that evidence provision may have been used strategically to reinforce a cohesive paradigm. In the malaria network, the odds of a provision tie were 2.77 times more likely (*θ* = 1.02; OR = 2.77, *p* < 0.05) if actors had research experience and were half as likely (*θ* = −0.671, OR = 0.51, *p* < 0.05) if they worked for a CSO/NGO compared to working for the government. As hypothesized, there was no evidence of reciprocity in any of the issues, suggesting that evidence was provided and requested in a hierarchical manner.

**Table 2:** **Parameter estimates (standard errors) of provision networks**

	**Child health**			**Malaria**			**HIV**		
	**Structural effects model**	**Attribute effects model**	**Full model**	**Structural effects model**	**Attribute effects model**	**Full model**	**Structural effects model**	**Attribute effects model**	**Full model**
*Structural effects*									
Edge intercept	−4.52 (0.96)*	−1.18 (0.70)*	−4.65 (1.83)*	−2.13 (0.32)*	−2.20 (0.44)*	−2.56 (0.52)*	−1.03 (0.37)*	−1.99 (1.03)*	−1.79 (1.14)
Triangle closure	2.81 (0.63)*	__	2.36 (0.78)*	0.74 (0.47)	__	0.78 (0.85)	0.382 (0.79)	__	0.405 (1.45)
Reciprocity	−5.10 (1.92)*	__	−3.52 (2.20)	−1.20 (1.15)	__	−1.71 (1.29)	0.007 (0.93)	__	0.318 (1.40)
Layering	5.15 (1.44)*	__	6.16 (1.73)*	3.23 (0.59)*	__	2.87 (0.63)*	2.29 (0.83)*	__	2.31 (0.98)*
*Attribute effects*									
Researcher	__	−0.23 (0.42)	−0.93 (0.98)	__	1.43 (0.37)*	1.021 (0.44)*	__	0.505 (0.56)	0.154 (0.60)
Civil society org.	__	No obs.	No obs.	__	−0.46 (0.32)	−0.671 (0.40)*	__	0.150 (0.46)	−0.120 (0.49)
Development partner org.	__	1.02 (0.45)*	0.31 (1.14)	__	−1.10 (0.67)	−0.813 (0.79)	__	3.37 (1.15)*	3.50 (1.16)*
Other	__	2.43 (1.19)*	5.04 (5.29)	__	0.81 (0.74)	0.217 (0.94)	__	2.10 (0.89)*	1.61 (1.04)
Manager/director	__	0.33 (0.35)	0.16 (0.80)	__	nf	nf	__	nf	nf
*Homophily*									
Researcher	__	nf	nf	__	nf	nf	__	nf	nf
Organization		nf	nf	__	nf	nf	__	nf	nf
AIC	233.7	179.5	176.4	345.3	369.1	342.8	210.1	191.6	193.1

*p < 0.05; nf = did not improve model fit.

#### Request models

Request ties were best fit in models combining structural and attribute effects (see Table 3 for full results). Layering, or the simultaneous existence of a request and a provision tie, remained the strongest predictor of tie formation in these models. Child health again had a significant coefficient for triangle closure (*θ* = 1.53; OR = 4.62, *p* < 0.05), meaning that the odds of tie formation were 4.62 times greater if the tie closed a triangle between three actors, conditional on the remainder of the model. Although this coefficient is not as large as in the provision network, request ties still seemed to occur to either strengthen or reinforce cohesion and shared norms in this case. As in the provision models, malaria actors were more likely to have request ties if they had research experience (*θ* = 1.73; OR = 5.64, *p* < 0.05), suggesting that this policy issue, in particular, had narrowly defined roles for who exchanged evidence. The malaria network demonstrated a significant negative effect of homophily within organizations (*θ* = −2.24; OR = 0.11, *p* < 0.05), meaning that actors were more likely to request research evidence outside of their organizations as compared to within, which runs counter to our hypotheses but presents a picture of interorganizational exchange of evidence for this case. The odds of forming a request tie were 3.36 times higher (*θ* = 1.85, OR = 3.36, *p* < 0.05) if an actor belonged to the ‘other’ organization category in the HIV network, a category which includes consultants and researchers. This is to be expected in a network that had high representation of CSO/NGO actors, who typically perceived a smaller role for research evidence as compared with lived experiences.

**Table 3:** **Parameter estimates (standard errors) of request networks**

	**Child health**			**Malaria**			**HIV**		
	**Structural effects model**	**Attribute effects model**	**Full model**	**Structural effects model**	**Attribute effects model**	**Full model**	**Structural effects model**	**Attribute effects model**	**Full model**
*Structural effects*									
Edge intercept	−4.00 (0.997)*	−1.47 (0.406)*	−4.67 (1.15)*	−3.66 (0.545)*	−2.64 (0.787)*	−4.19 (1.19)*	−3.37 (0.743)*	−5.83 (1.92)*	−6.74 (2.21)*
Triangle closure	1.49 (0.812)*	__	1.53 (0.848)*	1.26 (0.601)*	__	1.65 (2.88)	nf	__	nf
Reciprocity	−1.93 (1.49)*	__	−1.90 (1.62)	0.029 (1.28)	__	−1.43 (1.49)	1.98 (1.34)	__	−0.842 (2.14)
Layering	2.72 (1.12)*	__	2.96 (1.14)*	3.28 (0.604)*	__	3.18 (0.733)*	2.27 (0.832)*	__	2.31 (0.992)*
*Attribute effects*									
Researcher	__	0.088 (0.401)	0.652 (0.486)	__	1.90 (0.473)*	1.73 (0.688)*	__	1.70 (0.901)*	1.64 (1.01)
Civil society org.	__	nf	nf	__	−0.490 (0.498)	−0.313 (0.593)	__	1.30 (0.731)*	1.31 (0.788)
Development partner org.	__	nf	nf	__	−2.29 (0.979)*	−3.34 (1.41)*	__	nf	nf
Other	__	nf	nf	__	−0.407 (0.944)	−1.27 (1.21)	__	2.32 (0.953)*	1.85 (1.10)*
Manager/director	__	nf	nf	__	0.619 (0.379)	0.613 (0.478)	__	nf	nf
*Homophily*									
Researcher	__	nf	nf	__	nf	nf	__	nf	nf
Organization	__	nf	nf	__	−1.60 (0.663)*	−2.24 (0.933)*	__	nf	nf
AIC	183.4	208.7	183.5	310.6	334.7	306.9	162.7	171.6	DNC

*p < 0.05; nf = term did not improve model fit.

#### Are exchange relationships associated with the use of research evidence?

Based on analysis and coding of interview data, 43.0%, 34.6%, and 31.6% of policy actors in child health, malaria, and HIV domains, respectively, used research evidence during the policy processes to inform their professional decisions. Actively providing evidence was positively associated with the use of evidence across all issues (see Table 4). Receiving a request for research evidence (in-degree) was associated with use in the malaria domain, and sending a request (out-degree) associated with use in the user-fees domain. The statistical significance of the continuous degree variable suggests a dose–response effect; in other words, an actor is more likely to use evidence for each additional exchange he/she has. Multivariable models combining actors’ degree and their individual attributes demonstrated that degree was more predictive of research use than their attributes.

**Table 4:** **Univariate logistic regressions of actor degree on evidence use**

	**Child health**	**Malaria**	**HIV**
**Independent variable**	**Log odds (SE)**	**Log odds (SE)**	**Log odds (SE)**
Indegree (provision)	−0.336 (1.16)	0.896 (0.941)	−1.01 (1.16)
Outdegree (provision)	3.87 (1.51)*	Predicted perfectly	3.31 (1.34)*
Indegree (request)	0.223 (1.10)	1.86 (0.949)*	0.118 (1.05)
Outdegree (request)	1.79 (1.31)	1.40 (0.881)	3.31 (1.34)*
N	16	26	19

*p < 0.05.

## Discussion

This study illuminated the conditions under which evidence was provided and requested in three policy cases, and confirmed that evidence exchange is closely correlated to its use in health policymaking. Evidence provision and request ties were best predicted by structural factors, particularly layering (*i.e.*, actors are more likely to send ties when they complement existing ties), consistent with social network theories of the significant role of networks and structure in predicting individual-level behaviours. Some individual attributes mattered, particularly the role of research experience in the malaria domain, but should not be solely relied upon to design or target knowledge translation interventions. In terms of evidence use, network position—as judged by connectedness to others—predicted use better than any individual characteristic—not job level, not organizational affiliation, not experience as a researcher. This finding is consistent with other findings that show interpersonal relationships with researchers to be the best predictor of evidence use by policy-makers [12]. Taken together, these findings support taking a network lens when designing knowledge translation or evidence-informed policymaking interventions [48] and focusing on strategies that increase an actor’s capacity to provide and request evidence.

### Findings in relation to our hypotheses

Our findings were generally consistent with our hypotheses and clarify network theories that had not yet been tested in a low-income country policy setting. Only the child health network demonstrated a tendency for triangle closure. This network had the highest density of research provision and request ties and also had the highest proportion of actors who used research evidence to inform their decision-making, consistent with other studies that find the transfer of complex or tacit knowledge is aided by closed, cohesive networks that enable repeated exposure necessary for the synthesis and interpretation of complex ideas [22]. However, findings from linked studies suggest that while evidence was exchanged by dyads and used by actors in this network, the macro-level network used evidence ‘symbolically’ to justify pre-determined policy positions of certain actors [49]. The child health policy process advanced as part of a funding proposal process where the funders required that development partners had an equal seat at the table; respondents reported that most research evidence was disseminated strategically by development partners, typically to persuade hesitant government policy-makers. Thus, it is possible that active advocacy and persuasion during this policy process led to the observed triangle closure as opposed to pre-existing cohesiveness amongst actors in this domain. This finding is consistent with studies of information diffusion that contrast structural effects at dyadic and macro levels [34]. There is no question that actors can influence the shape of networks and their outcomes [50].

Ties were not reciprocated. Patterns of provision and request were hierarchical and unidirectional, as was expected for this context. Layering of the two relations was observed to a large degree, suggesting that these networks function instrumentally in that research is generally provided only when it is requested, and that most requests are realized. In considering these findings together, one can imagine a hierarchy of evidence flows, where requests flow one way and provisions flow the opposite direction, most often between the same individuals, but rarely will a pair reverse their roles as requesters and providers. Overall, evidence was provided more often than it was requested. This is consistent with the qualitative interviews where respondents suggested that certain actors, particularly development partners, provided unsolicited evidence.

We observed an absence of homophily, counter to our hypotheses. Homophily covariates did not improve model fit and were thus excluded, except in the case of the malaria request network where actors were less likely to exchange evidence if they belonged to the same organization. This finding reflects the malaria domain in Burkina Faso, where formal and informal institutional arrangements encourage exchanges between government policy-makers and research organizations. In the child health domain, the strategic dissemination of evidence by development partners overcame tendencies towards homophily. The finding that no single individual attribute seemed to drive tie formation was counter to our hypothesis, and to the implicit design of many knowledge translation interventions, but was also recently observed in a municipal public health department in Ontario [20].

### Strengths and limitations

This study is the first to empirically measure and model research evidence exchange in policy networks and provides important insights for evidence use in low-income countries. The finding linking frequency of exchanges with likelihood of use is likely generalizable across policy issues and jurisdictions. However, understanding how evidence is being used (and therefore why it is being exchanged) will require knowledge of specific issues and context.

Linked studies exploring the contextual factors surrounding evidence use in these cases add clarity to the motivations for evidence exchange and use; for example that dense exchange networks for child health were associated with symbolic use, that there was little motivation for evidence use in the malaria case, and that evidence was used instrumentally to change policy in the HIV case. Future examples of policy networks will enable the refinement of theories related to network-based markers of evidence use and exchange. Finally, this study is limited, as are most social network analyses, by challenges in collecting complete data on whole networks. Missing data may affect our results.

### Implications for policy and practice

In considering how to design network-based interventions for various settings, we suggest the aphorism: ‘know your network.’ Any knowledge translation intervention should begin with a baseline mapping of relevant policy network(s). While well-designed sociometric surveys remain the gold standard for network mapping, we acknowledge that sampling or ego-based approaches may still add-value above and beyond the status quo. Many local stakeholders have excellent intuition about their networks that can be harnessed to this end.

Deliberative dialogues could use this information to ensure that dialogue attendees are in strategic network positions—not just in strategic organizational positions. Dissemination of research evidence should include a message to share the evidence with a colleague or friend, and an encouragement to also request evidence from those same people. Ultimately, cultural shifts are needed to increase the perception that anyone can provide (or request) evidence; these shifts have occurred in other settings by building capacity and shifting incentives [17].

## Conclusion

This study explores the exchange and use of research evidence among policy actors in Burkina Faso. It is among the first of its kind of describe structural and attribute-related factors associated with exchange relationships among policy actors. Study findings suggest that while research exchange networks and their outcomes are highly issue-dependent, networks have a significant influence on knowledge exchange and use. Network variables—including the propensity to send complementary ties, to join sets of three actors, and overall connectedness—were more important than individual characteristics in predicting whether research evidence was provided or requested between actors, and were certainly more important in predicting an actors’ use of evidence. These findings can be leveraged to design knowledge transfer interventions which focus on facilitating or reinforcing exchange relationships.

## Additional files

Additional file 1:
**Consort diagram detailing this process.**
Additional file 2:
**Goodness-of-fit test results.**
