# Supplementary figures and images for: Exchanging and using research evidence in health policy networks: a statistical network analysis
Source: Implement Sci. 2014 Oct 30;9:126. doi: 10.1186/s13012-014-0126-8 (PMC4226903; doi:10.1186/s13012-014-0126-8)

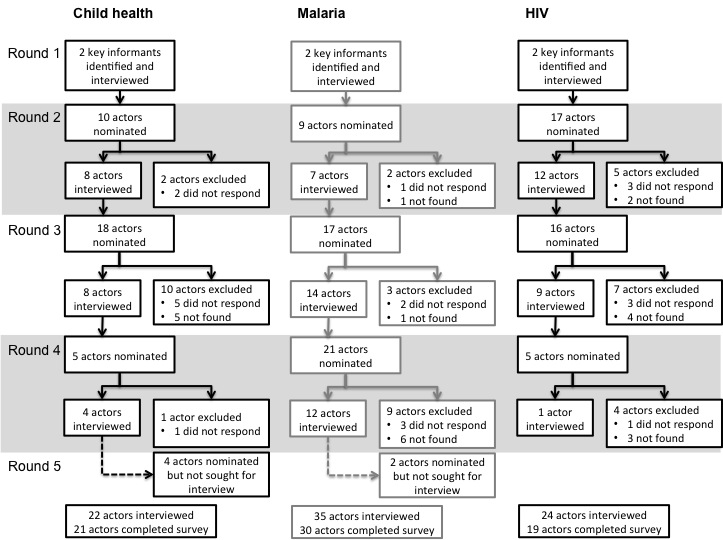

Supplement: Additional file 1: — Consort diagram detailing this process. [file 13012_2014_126_MOESM1_ESM.jpeg]

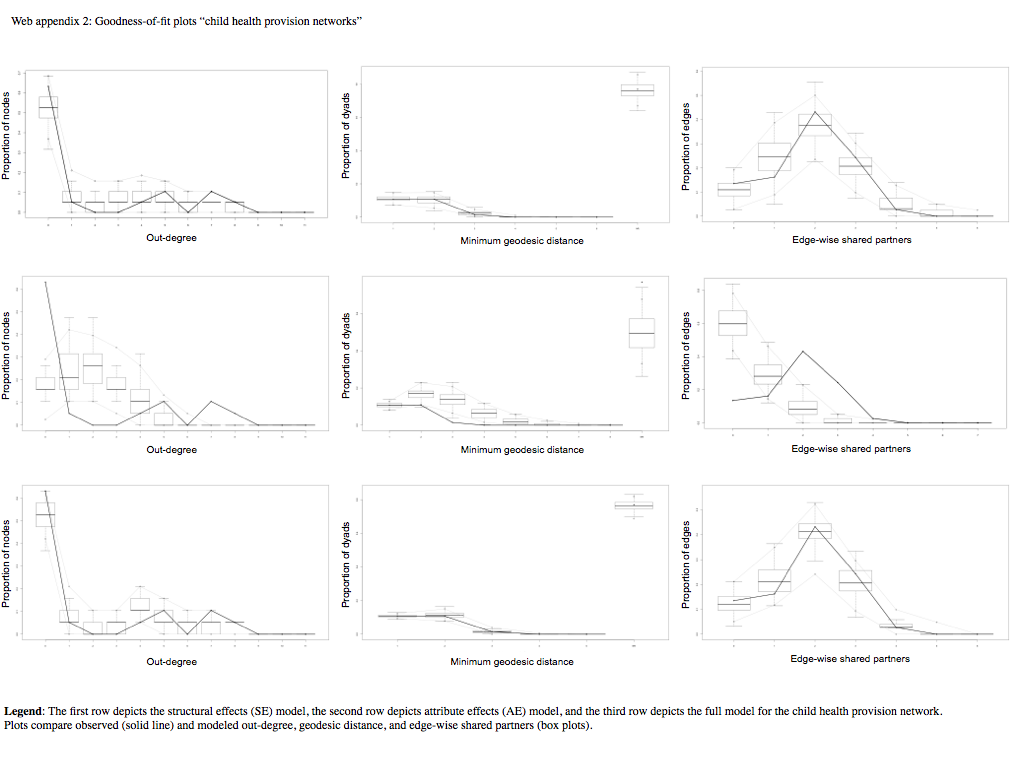

Supplement: Additional file 2: — Goodness-of-fit test results. [file 13012_2014_126_MOESM2_ESM.tiff]
